# The effect of addition *Eriobotrya japonica* L. marmalade on physicochemical, microbiological, and sensory properties of probiotic yogurts

**DOI:** 10.3389/fnut.2023.1151037

**Published:** 2023-03-30

**Authors:** Tamer Turgut, Abdulkerim Diler

**Affiliations:** ^1^Department of Food Processing, Technical Vocational School, Atatürk University, Erzurum, Türkiye; ^2^Department of Plant and Animal Sciences, Technical Vocational School, Atatürk University, Erzurum, Türkiye

**Keywords:** *Bifidobacterium*, *Eriobotrya japonica* L., probiotics, probiotic yogurt, yogurt texture

## Abstract

This study investigated the effects of loquat (*Eriobotrya japonica* L.) marmalade (LM) supplementation in probiotic yogurt during a 21 days storage period. In addition, the viability of *Bifidobacterium* and its effect on yogurt quality were investigated. Four types of yogurt, including plain yogurt (LM0) and yogurts with 5%, 10%, and 15% LM, were prepared. On days 1, 7, 14, and 21 of storage, physicochemical properties, microbial growth, and textural and sensory properties were investigated. The addition of LM to yogurt significantly affected the total dry matter, fat, pH, titratable acidity, syneresis, water holding capacity, and color parameters (L*, a*, b*). The addition of LM caused a decrease in L* (from 87.52 to 81.78) and an increase in a* values (from −35.42 to −30.14). Yogurts containing 10 and 15% LM demonstrated lower syneresis than control samples during storage. During storage, the pH of yogurts continuously decreased (*P* < 0.01). The viability of *Bifidobacterium* in yogurt was not affected by the LM addition. During storage, the viable count of *Bifidobacterium* ssp. decreased in all yogurt types. *Lactobacillus delbrueckii* ssp. bulgaricus decreased more than *Streptococcus thermophilus* did during storage. In all yogurt samples, coliform bacteria stayed below detectable concentrations. When a general evaluation was made by considering the physicochemical quality, sensory, and textural properties of all yogurt samples, it was revealed that LM-added yogurts can be produced and stored for 21 days.

## 1. Introduction

The majority of probiotic foods are milk-based, and their consumption has increased in recent years. Dairy foods containing probiotic bacteria play an important role as functional foods, and several studies have revealed their benefits to human health. Fermented milk and various yogurts are widely used and preferred for probiotic bacterial supplementation by customers worldwide, and their regular consumption has been shown to provide health benefits (1–3). Probiotics can be defined as live microbial mono- or mixed-culture dietary supplements that benefit human or animal hosts by improving the properties of native microbial flora (4, 5). Probiotics are live microorganisms that offer health benefits when consumed in food or supplements (6). *Lactobacillus* species are known for their antimicrobial and antiviral properties and play an important role in treating gastrointestinal disorders (7, 8). Probiotic species play a vital role in the improvement of lactose intolerance, inhibiting pathogens, lowering cholesterol, immune responses, prevention of intestinal and vaginal infections, against certain cancer varieties, food allergies, improving calcium absorption, immunostimulation and immunomodulation, constipation treatments, and SARS coronavirus (COVID-19) (5, 9, 10). It is now understood that intestinal microflora is closely related to human health (11, 12). However, adequate viable cells must be consumed regularly for the probiotic effect to occur in the consumer. Probiotic products must contain a minimum of 6–7 log CFUg^–1^ of viable cells of the probiotic microorganism (13).

*Eriobotrya japonica* L. is an evergreen tree in the Rosaceae family native to southeastern China that also grows in Korea, Japan, India, and other countries (14). Loquat has high medicinal value and has been used as a folk medicine for over 1000 years. Loquat extract has been used to counteract inflammation, cough, diabetes, chronic bronchitis, inflammation, cancer, and other health problems (15). Loquat can be eaten raw, but it is also used in processed foods such as jelly and jam (14). Consuming fruits and vegetables has been demonstrated in research to offer considerable protective health effects against chronic disease (16, 17). Consumption of fiber helps prevent obesity, atherosclerosis, colon cancer, and diabetes and also helps strengthen the natural gut flora (18). Loquat is rich in fiber and low in calories. The bioactive compounds in loquat have anti-cancer, anti-inflammatory, anti-diabetic, and anti-aging properties. The fiber content of loquat is 0.8–1.7 g/100 g per fruit (19).

This study aimed to investigate the potential benefits of incorporating loquat as a functional ingredient in probiotic fruit yogurts. Loquat is known for its high fiber content, which promotes the growth of probiotic bacteria and can contribute to better intestinal health. In addition, the use of loquat can enhance the nutritional value and taste of yogurt, potentially leading to increased consumption. We evaluated the physicochemical, sensory, and textural properties of the yogurts, along with their microbiological changes. A test panel assessment was conducted to evaluate the sensory properties of the different concentrations of loquat used in the yogurts (ranging from 5 to 15%). The goal of this study is to contribute to the existing literature on probiotic yogurt enriched with fiber-rich fruits, with the ultimate aim of promoting better consumer health.

## 2. Materials and methods

### 2.1. Material

The cow milk used for yogurt production was obtained from a small-scale dairy factory at Atatürk University (Erzurum, Turkey). The somatic cell count of the milk was 110.000 CFU/ml. Commercial probiotic yogurt cultures containing *S*. *thermophilus*, *L*. *bulgaricus*, and *Bifidobacterium* species (*Bifidobacterium longum*, *B*. *bifidum*, and *B*. *infantis*), labeled as Green Label ABY- 1, was supplied from Chr. Hansen (Istanbul, Turkey). The cultures were used in direct vat inoculation form and were inoculated following the manufacturer’s instructions. Fresh *Eriobotrya japonica* L. was purchased from local supermarkets in Erzurum. Orange-colored, plump, and fresh fruits were selected.

### 2.2. Preparation of loquat marmalade

Firstly, fresh, mature loquats were sorted and washed. The seeds and skin of the loquat were carefully removed, and the fruits were smashed with an Ultra Turrax homogenizer (Ika T25 Plus, Germany). An equal percentage of granulated sugar was incorporated into the fruit pulp. After the pulp was pasteurized at 90°C for 5 min, it was transferred to a sterile glass container and stored in the refrigerator until use.

### 2.3. Production of yogurts

Raw cow’s milk was concentrated by heating to 12% nonfat milk solids to increase total solids and cooled to 43 °C for final incubation. To prepare the primary culture, as recommended by the manufacturer, 50 units of DVS cultures were dissolved in 500 mL of milk, and 24 mL of this mixture was taken and added to 12 L of milk. The inoculated milk was divided equally into four batches. All batches were incubated for final fermentation at 43°C until pH reached 4.6. After incubation, the yogurt samples were cooled to 4°C and stored overnight. One group was reserved as control yogurt (LM 0), and the other three yogurt groups were mixed with LM at 5% (LM 5), 10% (LM 10), and 15% (LM 15). In the final step, the probiotic yogurt samples were filled into sterile 170 mL jars and stored at +4°C. The samples were subjected to physicochemical, microbiological, and sensory analyses on days 1, 7, 14, and 21.

### 2.4. Microbiological analysis

The yogurt samples (10 g) were aseptically weighed into a sterile Stomacher bag and homogenized in 90 mL of 1/4 Ringer’s solution (Merck, Germany) for 2 min to obtain a 10^–1^ dilution. Sequential dilutions were made with 1/4 Ringer’s solution up to 10^–6^ and then spread on the plates in duplicate. M17 agar (Merck, Germany) was used for counting *S. thermophilus*, and plates were incubated at 35–37°C for 48 h. Typical colonies (small, cream-colored colonies) were counted at the end of incubation. De Man, Rogosa, and Sharpe Agar (MRS) (Merck, Germany) and a CO_2_ atmosphere in anaerobic jars (Anaerocult C, Merck, Germany) at 37°C for 3 days were used to count *L*. *bulgaricus*. The bifidobacterial counts were determined using bifidobacterial-selective agar (BSM, Fluka). BSM plates were incubated under anaerobic conditions (Anaerocult C, Merck, Germany) at 37°C for 72 h (1). The BSM medium was prepared as follows: 0.116 g of BSM supplement (Fluka 83055) was dissolved in 10 mL of sterile water, added to sterilize warm BSM agar, and poured into petri dishes. BSM Agar was specifically selected for the enumeration of *Bifidobacterium* strains and inhibited *Lactobacillus* and *Streptococcus* strains. Plate Count Agar (PCA) (Merck, Germany) was used to enumerate the total viable aerobic mesophilic bacteria. The PCA plates were incubated at 30–32°C for 48 h. Violet Red Bile Agar (VRB; Merck, Germany) was used to count coliform bacteria, and the plates were incubated at 35–37°C for 24 h (20). Plates with 25–250 colonies were counted and expressed as colony-forming units (CFU) per gram of material.

### 2.5. Physical and chemical analyses

The total solids content of the yogurt samples was determined by drying the samples at 103°C to a constant weight, and the fat content was determined by the Gerber method according to Association of Official Agricultural Chemists [AOAC] (21). The amount of synergism present in the yogurt samples was determined using the method described by Bulca et al. (22). 25 g of yogurt samples were weighed and filtered for 2 h at 4°C *via* a funnel using filter paper (Whatman No. 1, UK). The following formula was used to determine syneresis:


$$Syneresis\left(\%\right)=\frac{w\,h\,e\,y\,v\,o\,l\,u\,m\,e}{i\,n\,i\,t\,i\,a\,l\,v\,o\,l\,u\,m\,e}\times 100$$

WHC was also determined using the method described by Bulca et al. (22). The yogurt samples (10 g) were centrifuged (4500 × *g* for 30 min at 4°C). The filtrates were weighed, and the WHC values were calculated according to the following formula:


$$WHC\left(\%\right)=\left[1-\frac{f\,i\,l\,t\,r\,a\,t\,e\,w\,e\,i\,g\,h\,t}{i\,n\,i\,t\,i\,a\,l\,w\,e\,i\,g\,h\,t}\right]\times 100$$

The pH of yogurt samples was determined using a pH meter (Hanna, pH 211, Portugal) after performing a 2-point calibration. The titratable acidity (TA) of yogurt samples was determined by mixing a 10 g sample with the same volume of distilled water and titrating with 0.1 N NaOH using phenolphthalein as an indicator. TA was expressed as the volume (mL) of NaOH consumed in the titration, and was calculated according to the following formula:


$$T\,A\%=\frac{V\,\left(m\,L\,N\,a\,O\,H\right)\times 0.9}{m}$$

Where m is the weight of the sample.

### 2.6. Colorimetric analyses

The color parameters were measured using a color meter (PCE XXM-20, PCE instrument), and the results were expressed using the CIELAB color system, with L*, a*, and b* values at illuminant D 65. The parameters were L*; 0–100 (black-white), a*; (−a*, +a*) (greenness, redness), and b*; (−b*, +b* (blueness, yellowness). Three replications were performed for each sample.

### 2.7. Sensory and texture analyses

The sensory assessment of the yogurt samples was performed on a sensory rating scale from one to nine (poor to very good) for all attributes described (odor, texture, flavor, syneresis, acidity, sweetness, and overall acceptability), as defined by Gürsel and Karacabey (23) and Roland et al. (24). All yogurt samples were served at 5°C to seven panelists selected from among non-smokers. The textural properties of the yogurts were assessed using a texture analyzer (TA-XT Plus; Stable Micro System Ltd, UK) fitted with a 500 g load cell. The yogurt samples were analyzed immediately after removal from the refrigerator (5°C). An aluminum extrusion cylinder probe (P25) with a diameter of 25 mm was used and adjusted to a penetration depth of 30 mm at a speed of 1.0 mm/sec. The P 25 probe speed was set to 1.0 mm/sec during the compression and release of the sample. Texture profile analysis (TPA) was used to calculate the basic textural parameters expressed as hardness, adhesiveness, springiness, cohesiveness, chewiness, and resilience values. The force-time curves were analyzed using Exponent Micro System software (v. 4.0.9.0). All measurements were performed in duplicate.

### 2.8. Statistical analysis

A general linear model and statistical analysis were used to assess microbiological, physicochemical, and sensory characteristics on days 1, 7, 14, and 21. All analyses were performed in duplicate. Data from this study were compared with Duncan’s multiple range test (*P* < 0.05) using SPSS 20.0.

## 3. Results and discussion

### 3.1. Measurement of pH and TA

**FIGURE 1:**
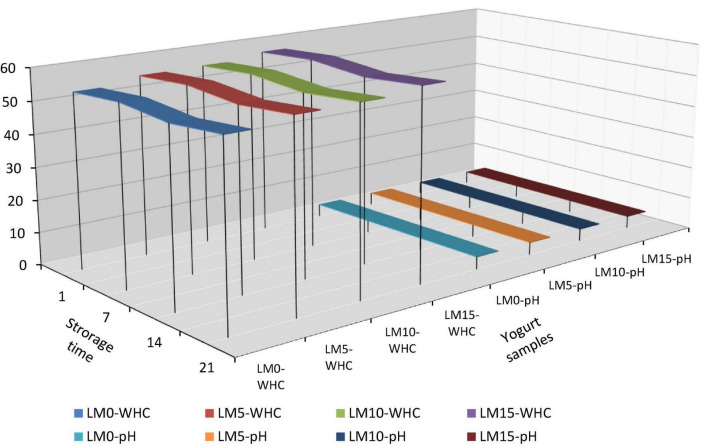
WHC and pH values of yogurt samples during storage.

The results of the physicochemical and microbiological analyses are presented in Table 1. The pH and TA values of yogurt samples were significantly affected by LM addition and storage time (*P* < 0.01). LM-supplemented yogurts showed higher pH values and lower acidity than control yogurts (Figure 1). The initial pH of the yogurt samples varied between 3.95 and 4.01. The mean pH of the yogurt samples ranged from 3.93 (LM0 and LM5) to 3.96 (LM15). The initial TA values of the yogurt samples varied between 1.197 and 1.355%, and the mean TA values for yogurt samples ranged from 1.197% for LM15 yogurt to 1.355% for the control yogurts (Table 1). Çakmakçı et al. (25) stated that the pH value of probiotic yogurts prepared with banana fruit ranged from 4.07 to 4.60 during the storage period. However, the pH values in our study were lower than those previously stated. Hossain et al. (26) reported that the natural acidity of fruit causes an increase in the acidity of fruit yogurt. Donkor et al. (27) stated that yogurt cultures were responsible for the increase in yogurt acidity during storage. Arslan and Bayrakci (28) found that the viability of yogurt bacteria is adversely affected by the sugar concentration. This data is compatible with that of our study. This can be explained by the antagonistic effect of sugar content on yogurt culture. Kumar and Kumar (29) reported that the TA acidity of probiotic fruit yogurts ranged from 0.45 to 0.71%. These values were higher than these values. In this study, the pH of all yogurts decreased and TA values increased during the 21 days of storage. This process is called post-acidification and influences the viability of yogurt bacteria. The decline in pH in yogurts during storage is known as “post-acidification” (1). The lowest TA value (1.229%) was observed on the 1st day for the LM15 yogurt, and the highest value (1.323%) was observed on the 21st day for the control yogurts (Table 1). This increase can be attributed to the multiplication of lactic acid bacteria and the generation of lactic acid during storage. Bakırcı and Kavaz (30) found similar results.

**TABLE 1:** Changes in physicochemical characteristics of yogurt samples during storage.

		Dry matter (%)	Fat (%)	Syneresis (%)	WHC (%)	Acidity (%)	pH
**Yogurt samples**							
LM0		14.092^a^	4.488^a^	33.310^a^	54.089^a^	1.355^a^	3.933^ab^
LM5		15.498^b^	4.075^b^	33.095^a^	56.081^b^	1.316^b^	3.926^a^
LM10		16.296^c^	3.400^c^	31.370^b^	56.385^b^	1.256^c^	3.950^bc^
LM15		17.834^d^	3.188^d^	31.530^b^	58.083^c^	1.197^d^	3.959^c^
**Storage time (days)**							
1		16.011	3.763	32.495	55.850^ab^	1.229^a^	3.974^a^
7		15.955	3.788	32.615	56.923^b^	1.274^b^	3.940^b^
14		15.582	3.825	32.860	55.256^a^	1.299^bc^	3.939^b^
21		16.172	3.775	31.335	56.610^b^	1.323^c^	3.915^c^
Source	D.F						
Yogurt samples	3	**	**	*	**	**	**
Storage time	3	NS	NS	NS	*	**	**
Error	24						
Total	31						

LM0 = natural yogurt (0% control); LM5 = yogurt with 5% fruit; LM10 = yogurt with 10% fruit; LM15 = yogurt with 15% fruit.

^a,b,c,d^Means in the same column without a common superscript differ (*P* < 0.05 or *P* < 0.01).

*Are significant at 0.05, **are significant at 0.01 probability levels. NS, not significant.

### 3.2. Syneresis and WHC

The release of serum, known as syneresis, is an important quality criterion for yogurt. A higher syneresis level indicates lower-quality yogurt (31). The highest syneresis value was found in the control (33.31%), and the lowest was in LM15 yogurt (31.53%) (Table 1). The decrease in pH stimulated syneresis in yogurt samples (Figure 1). The addition of LM decreased the syneresis values of the yogurt. However, the differences were not statistically significant (*P* > 0.05). In general, the syneresis values for all yogurt samples decreased during storage. The highest syneresis values (32.50%) were found on day one and the lowest values (31.335%) on day 21 (Table 2). However, the differences were not statistically significant (*P* > 0.05). The results showed that increasing the percentage of LM in yogurt resulted in lover syneresis. LM 15 yogurt had the lowest syneresis value compared with the other yogurts. Korkmaz et al. (32) reported that syneresis decreased in maca (*Lepidium meyenii*) powder and propolis-enriched yogurts. This result is consistent with the results of our study. WHC is generally referred to as the ability of food to retain natural or added water, and is important for the creation of food structure (33). There was a significant difference (*P* < 0.01) in WHC values between yogurt varieties. The WHC values of yogurts ranged from 54.09 to 58.083% (Table 2). The control yogurt had the lowest WHC values, followed by the LM-supplemented yogurts. WHC values increased proportionally with increasing LM percentages in yogurt samples. During the storage period, the WHC levels of the yogurts increased slightly but significantly (*P* < 0.05). These WHC values were lower than those reported by Karaca et al. (34) and similar to the results obtained by Bakırcı et al. (35).

**TABLE 2:** Color properties of the yogurt samples during storage.

		L*	a*	b*
**Yogurt samples**				
LM0		87.518^a^	−35.424^a^	7.415^a^
LM5		83.965^b^	−31.604^b^	8.175^b^
LM10		82.953^b^	−30.333^c^	8.674^c^
LM15		81.785^c^	−30.141^c^	8.975^c^
**Storage time (days)**				
1		83.983	−31.565	8.373
7		84.016	−31.940	8.234
14		83.925	−31.979	8.241
21		84.297	−32.019	8.392
Source	D.F.			
Yogurt samples	3	**	**	**
Storage time	3	NS	NS	NS
Error	88			
Total	95			

LM0 = natural yogurt (0% control); LM5 = yogurt with 5% fruit; LM10 = yogurt with 10% fruit; LM15 = yogurt with 15% fruit.

^a,b,c^Means in the same column without a common superscript differ (*P* < 0.01).

**Are significant at 0.01 probability levels.

NS, not significant.

L* a* b* symbols are used to represent color properties.

### 3.3. Color analysis

The color of fruit yogurts is a notable factor in consumer preferences and is often used to determine the sensory quality of yogurt. Table 2 shows the changes in instrumental color parameters for the control and LM-added yogurt samples. The addition of LM significantly affected (*P* < 0.01) the L*, a*, and b* values of the samples. The amount of LM decreased the L* value of yogurt but increased the a* and b* values. The highest L* value (87.52) was found in the control yogurt, and the highest a* and b* values (−30.14 and 8.97, respectively) were found in the LM15 yogurt. The L*, a*, and b* values of the yogurt samples were not affected by the storage time. In terms of color parameters, there was no significant difference between yogurt stored for one and 21 days (*P* > 0.05). Although a slight reduction in a* values was found during the storage period, these increases were not statistically significant. This can be explained by the fact that the natural pigments in the loquat were stable during the storage period and were not affected by the acidity of the yogurt. Ścibisz et al. (36) reported that the loss of color properties of the yogurt they produced with blueberries during storage depended on the fruit type and storage time.

### 3.4. Viable counts

Probiotic dairy products can provide a reasonably high number of bacteria while also providing additional advantages (10). Yogurt cultures that can survive in the small intestine are thought to be beneficial for human health (37). Table 3 shows the changes in the number of *S*. *thermophilus*, *L*. *bulgaricus*, *Bifidobacterium* ssp., and TAMB in the yogurts. The coliforms group remained below the detectable level in all analyzed yogurt samples (<2 log CFU/g). A small increase in *S*. *thermophilus* was observed during the storage period for all yogurt varieties. The decrease in pH during storage may be due to the increase in *S. thermophilus*, while the number of *L. bulgaricus* remained almost constant throughout the storage period. LM addition and storage time did not significantly affect (*P* > 0.05) the numbers of viable *S. thermophilus and L. bulgaricus*. Akın and Akın (38) reported a decrease in *S*. *thermophilus* and *L*. *bulgaricus* by about one log unit. Birollo et al. (39) reported a slight increase in *S*. *thermophilus* and *L*. *bulgaricus* in yogurts during a 60 days storage period, followed by a decrease. However, in our study, we found that the numbers of *S*. *thermophilus* and *L*. *bulgaricus* increased by only 0.2 and 0.1 log units, respectively. This result shows that a storage time longer than 20 days is a critical factor affecting the viability of *S*. *thermophilus* and *L*. *bulgaricus*. The difference between the TAMB counts of the control and LM yogurts was not significant (*P* > 0.05). The highest number of TAMB was found in the control yogurt group, and the lowest number was found in LM15 yogurts (Table 3). Çon et al. (40) stated that adding fruit to yogurt had no significant effect on his TAMB counts. This result is consistent with that of the present study. The number of TAMB in all yogurt samples increased slightly during the storage period, but these increases were not statistically significant (*P* > 0.05). The control yogurt and LM 5 yogurt had the highest number of *Bifidobacterium* and the LM 15 yogurt had the lowest. However, differences in bifidobacterial counts by yogurt type were not significant (*P* > 0.05) (Table 3). This result may be attributed to differences in the amount of sugar used in the preparation of the LM yogurts. Arslan and Bayrakçı (28) stated that sugar concentration negatively affects the viability of yogurt bacteria. The Bifidobacterial counts decreased in all yogurt types during storage, however, these decreases were not statistically significant (*P* > 0.05). It can be said that the number of *Bifidobacterium* in this study was well preserved. This can be explained by the fact that the applied heat treatment increased the dry matter and reduced the dissolved oxygen and redox potential. Dave and Shah (41) stated that the reduction in redox potential and dissolved oxygen promoted the growth of *Bifidobacterium*. The mean *Bifidobacterium* ssp. the number was found to be lower than both *L. bulgaricus* and *S. thermophilus* numbers of about 0.8 and 1.5 log units, respectively. Ranadheera et al. (42) reported similar results. Çakmakçı et al. (25) stated that food containing probiotic bacteria should have a minimum recommended content above 6 log CFU/g for optimal therapeutic effect. In this study, although the number of *Bifidobacterium* decreased during storage, all yogurt samples retained their probiotic characteristics throughout the storage period.

**TABLE 3:** Changes in microbiological characteristics of yogurt samples during storage (log CFU/g).

		*L. bulgaricus*	*S. thermophilus*	TAMB	*Bifidobacterium* ssp	*Coliform*
**Yogurt samples**						
LM0		7.841	8.675	8.429	7.336	<2
LM5		7.924	8.774	8.458	7.391	<2
LM10		7.910	8.592	8.359	7.291	<2
LM15		7.934	8.586	8.319	7.190	<2
**Storage time (days)**						
1		7.822	8.524	8.331	7.338^ab^	<2
7		8.158	8.770	8.478	7.412^a^	<2
14		7.852	8.576	8.403	7.253^ab^	<2
21		7.777	8.757	8.352	7.205^b^	<2
Source	D.F					
Yogurt samples	3	NS	NS	NS	NS	NS
Storage time	3	NS	NS	NS	*	NS
Error	24					
Total	31					

LM0 = natural yogurt (0% control); LM5 = yogurt with 5% fruit; LM10 = yogurt with 10% fruit; LM15 = yogurt with 15% fruit.

^a,b^Means in the same column without a common superscript differ (*P* < 0.05).

*Are significant at 0.05 probability levels.

NS, not significant.

L* a* b* symbols are used to represent color properties.

### 3.5. Textural and sensory analysis

**FIGURE 2:**
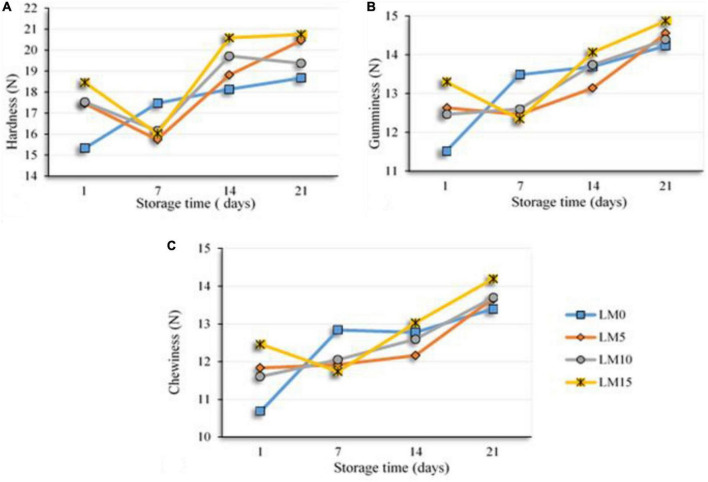
Changes in textural parameters of the yogurt samples during storage hardness **(A)**, gumminess **(B)**, and chewiness **(C)**.

**FIGURE 3:**
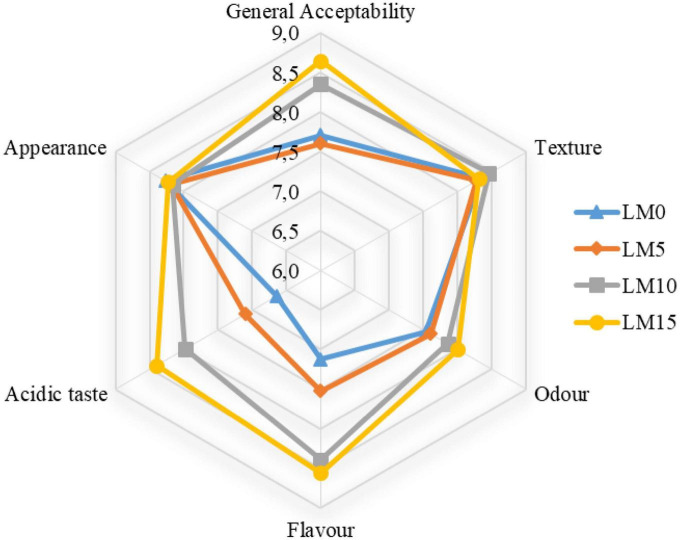
Sensory scores of yogurt samples.

The textural properties of fermented milk products, such as yogurt, are one of the main criteria that determine their acceptance by consumers (43). Table 4 shows the changes in the texture parameters of the yogurts during storage. The addition of LM to yogurt samples increased textural properties, such as hardness, gumminess, and chewiness, and decreased adhesiveness, cohesiveness, and resilience. However, these differences were not statistically significant (*P* > 0.05). All texture scores, except for the adhesiveness of LM 10 and LM15 yogurt samples, were higher than those of the control yogurt. There was a significant difference (*P* < 0.01) between textural properties and storage time. As expected, the scores of the hardness, gumminess, and chewiness of the yogurts increased significantly during the storage, and the differences were significant (*P* < 0.01) from day 14 (Figure 2). Some researchers have reported that long storage periods affect textural properties (such as hardness, firmness, and gumminess (18, 43). Najgebauer-Lejko et al. (44) stated that the textural properties of vegetables added to yogurts, such as hardness, gumminess, and cohesiveness, were insignificant. These results were consistent with our findings. Sensory analysis, especially taste and flavor evaluation, is a key attribute that plays an important role in general acceptance Falah et al. (45). Figure 3 shows the sensory evaluation results of the yogurt samples. The addition of LM increased the flavor, odor, acidic taste, and overall acceptability of yogurt samples. The appearance scores were slightly lower in the LM-supplemented yogurt samples than in the control group, but the difference was not significant (*P* > 0.05). The acidic taste and aroma scores of the LM-supplemented yogurts differed from each other and also from the control sample depending on the LM ratio. The highest acidic taste scores were obtained for LM15, LM10, and LM5 yogurts. Similarly, the highest general acceptability scores were obtained in the LM15 and LM10 yogurts (Figure 3). Sensory evaluation scores, including appearance, flavor, acidic taste, and overall acceptability scores, tended to decrease in the yogurt samples during storage. As predicted, acidic taste scores steadily decreased with storage. As yogurt is a living product, the bacterial content in yogurt causes an increase in the amount of lactic acid (Table 1). However, after the 14th day of storage, there was not much change in the acidic taste scores. Yogurts containing 15% LM were generally preferred by the panelists over all other yogurt samples.

**TABLE 4:** Textural properties of yogurt samples during storage.

		Hardness (N)	Adhesiveness (N.s)	Springiness (–)	Cohesiveness (–)	Gumminess (N)	Chewiness (N)	Resilience (–)
**Yogurt samples**								
LM0		17.406	−30.674	0.939	0.761	13.233	12.426	0.206
LM5		18.123	−31.540	0.940	0.736	13.199	12.397	0.201
LM10		18.197	−33.030	0.939	0.737	13.301	12.488	0.191
LM15		18.955	−34.320	0.942	0.728	13.649	12.855	0.183
**Storage time (days)**								
1		17.193^a^	−31.416	0.934	0.727	12.479^a^	11.648^a^	0.189
7		16.353^a^	−31.526	0.954	0.781	12.723^a^	12.138^ab^	0.227
14		19.318^b^	−32.549	0.925	0.711	13.662^b^	12.642^b^	0.167
21		19.817^b^	−33.073	0.947	0.743	14.517^c^	13.740^c^	0.197
Source	D.F							
Yogurt samples	3	NS	NS	NS	NS	NS	NS	NS
Storage time	3	**	NS	NS	NS	**	**	NS
Error	24							
Total	31							

LM0 = natural yogurt (0% control); LM5 = yogurt with 5% fruit; LM10 = yogurt with 10% fruit; LM15 = yogurt with 15% fruit. ^a,b,c^Means in the same column without a common superscript differ (*P* < 0.01).

**Are significant at 0.01 probability levels.

NS, not significant; N, Newton; N.s, Newton sec.

## 4. Conclusion

A novel probiotic fruit yogurt has been developed to deliver health benefits through probiotic bacteria to the human host. The present study reveals that the incorporation of 15% LM had a remarkably positive impact on the sensory and textural properties of the yogurt. However, the effect was uncertain with the use of 5% LM. Therefore, it can be concluded that a 10% LM ratio can serve as the acceptable threshold limit for optimal sensory and textural properties of loquat fruit yogurt.

The results of this study demonstrate that neither the addition of LM nor 21 days storage has any effect on the vitality of probiotic bacteria in yogurt; this is only affected after 21 days of storage. All yogurt types preserved their probiotic properties until the expiration of the storage period. Changes in pH values were compatible with the WHC and syneresis values. The addition of LM caused an increase in a* and b* values, but the values changed harmoniously according to the color of the fruit. Determining the number of probiotic bacteria in fermented foods like yogurt is difficult because of the presence of other lactic acid bacteria, however, in this study, the BSA medium was found to be effective for the enumeration of *Bifidobacteria*. The addition of LM improved the flavor, odor, acidic taste, and overall acceptability scores. This concludes that loquat is a suitable choice for probiotic yogurt production in terms of its sensory properties.

## Data availability statement

The original contributions presented in this study are included in the article/supplementary material, further inquiries can be directed to the corresponding author.

## Author contributions

TT prepared and revised the manuscript, methodology, and project management. AD provided the feedback during writing, mentoring, and project management. Both authors contributed to the manuscript and approved the submitted version.
